# A UPLC-MS/MS Method for Qualification of Quercetin-3-*O*-β-D-glucopyranoside-(4→1)-α-L-rhamnoside in Rat Plasma and Application to Pharmacokinetic Studies

**DOI:** 10.3390/molecules18033050

**Published:** 2013-03-07

**Authors:** Xin Yao, Guisheng Zhou, Yuping Tang, Zhenhao Li, Shulan Su, Dawei Qian, Jin-Ao Duan

**Affiliations:** Jiangsu Key Laboratory for High Technology of TCM Formulae Research, Nanjing University of Chinese Medicine, Nanjing 210046, China

**Keywords:** quercetin-3-*O*-β-D-glucopyranoside-(4→1)-α-L-rhamnoside, pharmacokinetics, UPLC-MS/MS

## Abstract

A sensitive and accurate ultra-performance liquid chromatography coupled with triple quadrupole mass (UPLC-MS/MS) method was developed for the determination of quercetin-3-*O*-β-D-glucopyranoside-(4→1)-α-L-rhamnoside (QGR) in rat plasma using rutin as internal standard. Chromatographic separation was achieved on a Acquity BEH C_18_ column (100 mm × 2.1 mm, 1.7 μm) with a gradient elution of acetonitrile and 0.10% formic acid (v/v) at a flow rate of 0.4 mL/min. QGR and rutin were detected using electrospray negative ionization mass spectrometry in the multiple reaction monitoring (MRM) mode. The method demonstrated good linearity and did not show any endogenous interference with the QGR and rutin peaks. This method was successfully applied to a pharmacokinetic study of QGR in rats after intravenous (20 mg/kg) and oral (40 mg/kg) administration, and the results showed that the compound was poorly absorbed, with an absolute bioavailability of approximately 3.41%.

## 1. Introduction

The dried leaves of *Ginkgo biloba* L. have been used as herbal remedies in China, and now their extracts are one of the most widely used herbal products and/or dietary supplements in the World, popularized for its alleged tonic effects and possible curative and restorative properties [1,2,3]. *G. biloba* leaves are rich in flavonol glycosides, terpene lactones, biflavones, and proanthocyanidins, of which the flavonol glycosides have received by far the most attention [4,5,6,7]. Quercetin-3-*O*-β-D-glucopyranoside-(4→1)-α-L-rhamnoside (QGR) was one of the important flavonol glycosides in *G. biloba* leaves, which have a variety of biological effects, such as scavenging free radicals [8,9], inhibiting the inflammatory response [10,11] and apoptotic regulation [12]. Therefore, pharmacokinetic studies of QGR on the basis of qualified bio-analytical methods are necessary to illustrate the mechanism of action.

To our knowledge, there are no published analytical methods available for the quantification of QGR in biological fluids. The aim of this present work was to develop an accurate and sensitive UPLC-MS/MS method for the quantification of QGR in rat plasma. The developed method was validated and then applied to a pharmacokinetic study of QGR after intravenous (20 mg/kg) and oral (40 mg/kg) administration QGR in rats.

## 2. Results and Discussion

### 2.1. Optimization of the Chromatographic Conditions

**Figure 1:**
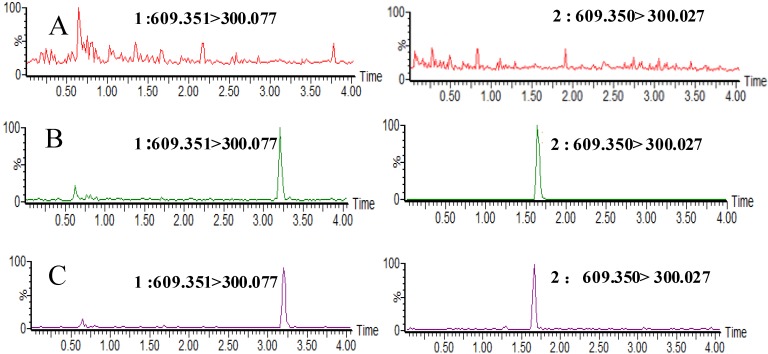
Representative chromatograms for QGR (**1**) and IS (**2**) in (**A**) rat blank plasma; (**B**) blank rat plasma sample spiked with QGR (740 ng/mL) and IS (500 ng/mL); (**C**) plasma sample collected at 30 min after intravenous administration of 20 mg/kg QGR.

To select a proper transition for the MS/MS detection of the analyte, QGR and IS were examined separately in direct infusion mode and at least two precursor/product ion pairs for each analyte were presented. Then, according to the quantitative results, the highest sensitivity and specific ion pairs were selected for the MRM determination. Once the most appropriate precursor/product ion pairs had been determined, the values of cone voltage and collision energy were optimized using the IntelliStart software. All the MRM transitions and parameters applied in the study are listed in Table 1. The results indicated that MRM mode had more advantages in the quantification of low content analysis. Representative chromatograms are shown in Figure 1.

**Table 1:** The molecular weight (MW), MRM transitions, cone voltage, collision energies, retention times (Rt) and ion mode of QGR and IS.

Compds.	MW	MRM transitions	Cone voltage (V)	Collision energies (eV)	Rt (min)	Ion Mode
IS	610	609.350 > 300.027	52	34	1.64	ESI^−^
QGR	610	609.351 > 300.077	50	34	3.23	ESI^−^

### 2.2. Method Validation

#### 2.2.1. Selectivity and Specificity

In the present study, the specificity and selectivity were examined using independent plasma samples from different rats. Figure 1 shows a typical chromatogram for the drug-free plasma (Figure 1A), drug-free plasma spiked with QGR and IS (Figure 1B) and an *in vivo* rat plasma sample after intravenous administration of QGR (Figure 1C). As shown in Figure 1, there is no significant interference from plasma found at retention times of either QGR or the IS. The retention time of QGR and the IS were approximately 3.23 and 1.64 min, respectively. The results indicated that the method exhibited good specificity and selectivity and was applied to plasma samples for the pharmacokinetic study.

#### 2.2.2. Matrix Effect

The matrix effect for the analyzed compound was evaluated in this paper by the ratio of response of spiked bio-samples to that of corresponding methanolic samples. No significant matrix effect was observed in control disposed bio-samples medium with the resulted ratios ranged from 1.05 to 1.17 in plasma and showed less than 20% variation.

#### 2.2.3. Extraction Recovery

The extraction recovery was determined in six replicates by comparing the peak areas of the extracted plasma at 14.8, 740 and 14,800 ng/mL with those obtained from the direct injection of standard solutions without preparation at the same concentrations. The extraction recoveries of QGR were 76.8 ± 4.6%, 77.1 ± 3.8% and 74.7 ± 4.5% for QC samples at the concentrations of 14.8, 740 and 14,800 ng/mL, respectively. All the data are summarized in Table 2, and the extraction recovery of the IS was 75.4 ± 3.7%. The recovery of the determination of QGR and IS in rat plasma was consistent, precise and reproducible.

**Table 2:** Extraction recovery of QGR in rat’s plasma (mean ± SD, *n* = 6).

Spiked plasma concentration (ng/mL)	Measured concentration (ng/mL)	Extraction recovery (%)	RSD (%)
14.8	11.3 ± 0.6	76.8 ± 4.6	5.8
740.0	570.5 ± 7.1	77.1 ± 3.8	6.7
14,800.0	11,055.6 ± 21.4	74.7 ± 4.5	4.5

#### 2.2.4. The Lower Limit of Detection (LLOD), Quantification (LLOQ) and the Linearity

The LLOD of the QGR assays demonstrated as 0.36 ng/mL (S/N ≥ 3), and the LLOQ was 1.25 ng/mL. At LLOQ, the accuracy was within ±7.8%, and the precision was less than 6.1%. The calibration curves ranged from 1.48 to 74,000 ng/mL using seven calibration standards. The regression equation for calibration curves in plasma was y = 0.7164x + 0.0981 (*n* = 5), where y is the peak-area ratio [(peak area of analyte)/(peak area of IS)] versus concentration, and *x* is the concentration of QGR. The correlation coefficient (*r*^2^) was ≥0.9989 for the calibration curves. The method was found to be sufficiently sensitive for the determination of the pharmacokinetic analysis of QGR in rats.

#### 2.2.5. Precision and Accuracy

The precision and accuracy data for intra- and inter-day plasma samples are presented in Table 3. The assay values for both occasions (intra- and inter-day) were found to be within the accepted variable limits. The intra- and inter-day precisions ranged 4.6 to 8.1% and 6.7 to 10.4%, respectively. The accuracy derived from QC samples ranged 2.8 to 7.3% and 3.9 to 4.6%, respectively. The data indicated that the present method has a satisfactory accuracy, precision and reproducibility.

**Table 3:** Precision and accuracy for the analysis of QGR in rat’s plasma (*n* = 5 days, six replicates per day).

Spiked concentration (ng/mL)	Intra-day			Inter-day			
	Measured concentration (ng/mL)	Precision (RSD, %)	Accuracy (RE, %)		Measured concentration (ng/mL)	Precision (RSD, %)	Accuracy (RE, %)
14.8	15.1 ± 0.7	8.1	7.3		15.2 ± 0.5	10.4	4.5
740.0	736.7 ± 3.4	4.6	−3.4		744.6 ± 4.7	6.7	4.6
14,800.0	14,857 ± 41.2	6.9	2.8		14,841 ± 47.8	7.7	3.9

#### 2.2.6. Stability

QC samples at three concentrations were analyzed in six replicates for studying the possible conditions to which the samples might be exposed during storage and handling. It was found that QGR was stable in rat plasma after being stored at room temperature for 4 h, after repeated three freeze-thaw cycles and after being stored at −80 °C for 30 days. In addition, the treated samples were found to be stable in the autosampler for a period of 24 h, and the results were found to be within the assay variability limits during the entire process. All results of the stability tests are summarized in Table 4.

### 2.3. Pharmacokinetic Study

**Figure 2:**
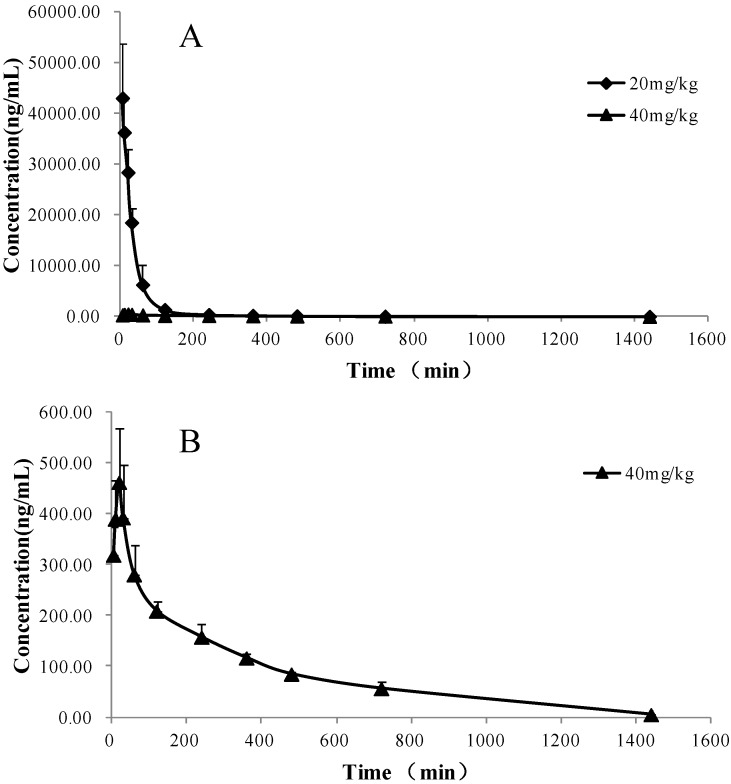
(**A**) Mean plasma concentration-time profiles of QGR determined by UPLC-MS/MS method after intravenous (20 mg/kg) and oral (40 mg/kg administration QGR to rats; (**B**) Mean plasma concentration-time profiles of QGR after oral (40 mg/kg) administration QGR to rats. Each point represents mean ± SD (*n* = 3).

This new developed method was applied to determine the plasma concentration of QGR in rats following intravenous (20 mg/kg) and oral (40 mg/kg) administration. The sensitivity and specificity of the assay were found to be sufficient for accurately characterizing the plasma pharmacokinetics of QGR in rats. The mean plasma concentration-time profiles of QGR after the two doses are illustrated in Figure 2 and its estimated pharmacokinetic parameters are presented in Table 5. The oral bioavailability (F) of QGR was calculated to be 3.41 ± 1.21% with an elimination half-life (t_1/2_) value of 236.87 ± 28.59 min. The potential hydrolysis in the gastrointestinal tract, poor permeability through the intestinal epithelial membrane and first-pass effect in the liver might be responsible for the low bioavailability of these compounds including QGR.

**Table 4:** Stability of QGR in rat plasma (*n* = 6).

Storage conditions	Concentration (ng/mL)		RSD (%)	RE (%)
	Spiked	Measured (mean ± SD)		
At room temperature for 4 h	14.8	14.7 ± 0.4	4.1	-2.5
	740.0	748.3 ± 3.1	4.6	2.8
	14,800.0	14,825 ± 20.5	6.1	4.5
After three freeze/thaw cycles in plasma	14.8	15.2 ± 0.6	4.7	4.1
	740.0	734.7 ± 6.3	7.5	−3.7
	14,800.0	14,822 ± 24.2	3.6	4.7
In the auto-sampler for 24 h	14.8	15.3 ± 0.8	2.7	2.6
	740.0	751.4 ± 8.7	2.7	3.9
	14,800.0	14,835 ± 28.9	3.2	6.8
Long-term stability (at −80 °C for 30 days)	14.8	15.4 ± 1.1	8.5	4.1
	740.0	761.7 ± 20.4	5.6	8.6
	14,800.0	14,871 ± 71.2	7.8	10.4

**Table 5:** Main pharmacokinetic parameters of QGR in rats determined after intravenous and oral administration (*n* = 3, mean ± SD).

PK parameters	Unit	Intravenous	Oral
t_1/2_	min	118.89 ± 5.65	236.87 ± 28.59
AUC(0_→_t)	μg/mL × min	1,775.96 ± 36.92	120.81 ± 11.38
AUC(0_→_∞)	μg/mL × min	1,790.24 ± 37.53	122.14 ± 16.15
Tmax	min	-	20
Cmax	ng/mL	-	495.69 ± 58.36
F	%	-	3.41 ± 1.21

## 3. Experimental

### 3.1. Chemicals and Reagents

**Figure 3:**
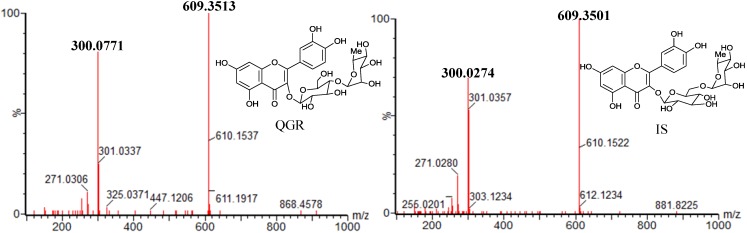
Full-scan product ion spectra of [M−H]^−^ ions and fragmentation schemes for QGR (C_27_H_30_O_16_, MW = 610) and IS (C_27_H_30_O_16_, MW = 610).

QGR (Figure 3) was previously isolated and identified from *G. biloba* leaves in our laboratory, and identified by ^1^H-NMR, ^13^C-NMR, MS and UV spectra. The purity of QGR was >98%, determined by HPLC analysis. The internal standard (IS) rutin (Figure 3) was provided by the National Institute for the Control of Pharmaceutical and Biological Products (Beijing, China). Methanol and Acetonitrile were HPLC-grade from Merck (Darmstadt, Germany), and deionized water (H_2_O) was purified by a super-purification system (Eped Technology Development Co., Ltd., Nanjing, China). Other reagent solutions were of analytical grade (Sino pharm Chemical Reagent Co., Ltd., Shanghai, China).

### 3.2. UPLC-MS/MS Instrument and Conditions

Determinations was performed on a Waters Acquity UPLC system (Waters Corp., Milford, MA, USA), consisting of a binary solvent delivery system, and an auto-sampler. A Waters Acquity BEH C_18_ column (100 mm × 2.1 mm, 1.7 μm) was used for all the analyses. The mobile phase consisted of A (0.1% formic acid, v/v) and B (acetonitrile), and was used in gradient elution. UPLC linear gradient conditions were: 0–4 min, 80–70% A. The column equilibrated was two minutes before the next injection. The flow rate of the mobile phase was 0.4 mL/min. The column temperature and injection volume were set at 30 °C and 1 μL, respectively. All the analyses were operated using MassLynx^TM^ XS Software.

Mass spectrometry detection was performed using a Xevo^TM^ Triple Quadrupole MS (Waters) equipped with an electrospray ionization (ESI) source. The parameters in the source were set as follows: The desolvation gas flow rate set to 1,000 L/h at a temperature of 550 °C, the cone gas flow rate set at 50 L/h and the source temperature at 150 °C. The capillary voltage set to 3,000 V. The analyte detection was performed by using multiple reaction monitoring (MRM). The cone voltage was set depending upon each specific MRM for each compound. Data was collected in MRM mode by screening parent and daughter ions simultaneously. Dwell time was automatically set by the software. All ESI and MS parameters were optimized individually for each target compound and were listed in Table 1.

### 3.3. Preparation of Calibration Standards and Quality Control Samples

Stock solutions of QGR and rutin (IS) were prepared both in methanol at a concentration of 0.148 mg/mL and stored at 4 °C. A series of working standard solutions of QGR ranging from 14.8 to 740,000 ng/mL and an IS solution at 500 ng/mL were prepared by diluting their stock solutions with the mobile phase. All the solutions were kept at 4 °C and were brought to room temperature before use. The plasma calibration standards of QGR were prepared as follows: 10 μL of the working solution was evaporated to dryness by a gentle stream of nitrogen, and then 100 μL of blank rat plasma was added to obtain the concentrations of 1.48, 14.8, 148, 740, 1480, 14,800 and 74,000 ng/mL. Quality control (QC) samples were prepared in the same way as the calibration samples, representing low, middle and high concentrations of QGR in plasma at 14.8, 740 and 14,800 ng/mL, respectively.

### 3.4.Sample Preparation

Rat plasma samples (100 μL) were pipetted into the 2 mL Eppendorf tubes, followed by IS (10 μL) and methanol (290 μL). The mixture was mixed for 3 min by a vortex before each sample was centrifuged (12,000 rpm) for 5 min, the supernatant was transferred and evaporated to dryness at 37 °C under a gentle stream of nitrogen. The residue was reconstituted in 100 μL of the mobile phase and an aliquot of 1 μL was injected for UPLC-MS/MS analysis.

### 3.5. Method Validation

A full method validation was performed according to the Food and Drug Administration (FDA) Bio-analytical method validation by evaluating selectivity, linearity, lower limit of quantitation (LLOQ), intra- and inter-day precisions and accuracy, recovery, matrix effect, and stability.

#### 3.5.1. Selectivity

To investigate whether or not endogenous constituents interfered with the assay, six different blank rat plasma samples were assessed on the potential interferences at the LC peak region for the analyte and IS using the proposed procedure and UPLC-MS/MS conditions.

#### 3.5.2. Linearity

The calibration curve was acquired by plotting the ratio of sum of peak area of QGR to that of IS against the nominal concentration of calibration standards. The standard curve was fitted to linear regression (y = a*x* + b) using 1/*x* as the weighting factor. Blank plasma samples were analyzed to confirm the absence of interferences but were not used to construct the calibration function. The concentrations of analytes in test samples calculated by the regression parameters from the calibration curves.

#### 3.5.3. Limit of Detection and Lower Limit of Quantification

The lower limit of detection (LLOD) of the MS analysis was defined as the analyte concentration in the plasma after the sample cleanup method that corresponds to three times the baseline noise (S/N ≥ 3). The lower limit of quantification (LLOQ) of the assay was assessed as the lowest concentration on the calibration curve that could be quantitatively determined with an acceptable precision less than 20% and an accuracy within ±20%, which was established based on six replicates independent of the QC samples.

#### 3.5.4. Precision and Accuracy

The intra- and inter-day precision and accuracy, defined as the relative standard deviation (RSD), were evaluated by analyzing QC samples at three different concentrations in six replicates on the same day and three consecutive validation days.

#### 3.5.5. Extraction Recovery and Matrix Effect

The extraction recoveries of QGR from rat plasma at three QC levels following protein precipitation were measured individually by comparing the mean peak areas obtained from six plasma samples spiked with the analytes prior to extraction with those added after extraction. The ratio gives the percentage recovery. The extraction recovery of the internal standard was determined in a similar way using the medium concentration of the QC as a reference. Matrix effects for the compounds were determined and calculated by the ratio of the response of methanol precipitated-blank bio-samples spiked with mixed stock standard solution over the response of corresponding mixed standard samples prepared by dilution in methanol.

#### 3.5.6. Stability

QC plasma samples at three concentrations were subjected to analysis using the conditions given below. The short-term stability was assessed by analyzing QC plasma samples kept at room temperature for 4 h. The autosampler rack stability was determined by analyzing the extracted QC plasma samples kept in an autosampler at 4 °C for 24 h. The freeze-thaw stability was investigated after three freeze (−20 °C)-thaw (room temperature) cycles. The long-term storage stability was assayed using QC plasma samples after storage at −80 °C for 30 days. The concentrations following storage were compared with those of freshly prepared samples of the same concentrations.

### 3.6. Pharmacokinetic Study in Rats

Six male Sprague-Dawley rats (weighing 220 ± 10 g, 8 weeks) were purchased from Shanghai Slac Laboratory Animal Co. Ltd (Shanghai, China) under license number SCXK (Shanghai) 2007–0005 and divided into two groups at random (*n* = 3 rats/group). All experimental procedures were approved by the Animals Ethics Committee of Nanjing University of Chinese Medicine. Polyethylene cannulas were implanted in the femoral vein 2 days before the experiment while the rats were anesthetized with an intraperitoneal dose of chloral hydrate at 350 mg/kg. The rats were fasted for 12 h before experiments with the exception of free access to water. The dosing solution with QGR concentration of 15.4 mg/mL was prepared by dissolving appropriate amount of QGR in normal saline solution and then given to rats at a single oral dose (40 mg/kg) by gastric gavage and intravenous dose (20 mg/kg) by rapid injection via the catheter. After intravenous administration of 20 mg/kg QGR through tail vein, aliquots of 0.50 mL blood samples were collected in sodium heparinized polyethylene tubes at different time intervals post-dosing (5, 10, 20, 30, 60, 120, 240, 360, 480, 720 and 1440 min). After oral administration, aliquots of 0.50 mL blood samples were collected in heparinized polyethylene tubes at different time intervals post-dosing (5, 10, 20, 30, 60, 120, 240, 360, 480, 720 and 1440 min). Heparinized blood was centrifuged at 12,000 rpm at room temperature for 5 min to obtain plasma, which was stored at −80 °C until analysis. All the data was processed by non-compartmental analysis using the DAS 2.0 package (Mathematical Pharmacology Professional Committee of China, Shanghai, China). The maximum plasma concentration (C_max_) and the time to reach C_max_ (T_max_) were directly obtained from the experimental data. The area under the plasma concentration-time curve (AUC) from time zero to the last quantifiable time point (AUC_0→t_) and from time zero to infinity (AUC_0→∞_) were estimated using the log-linear trapezoidal rule. Absolute bioavailability (F) was calculated based on the AUC_0→∞_ obtained after oral and intravenous administration at the equivalent dose.

## 4. Conclusions

A novel, simple and sensitive LC-MS/MS method has been developed for quantification of QGR in rat plasma. The method has been successfully applied in the quantification and pharmacokinetic study of QGR in rats after intravenous and oral administration with excellent sensitivity, good linearity of responses, and high precision and accuracy. The preliminary pharmacokinetic behavior of QGR was firstly elucidated.

## References

[1] Van Beek T.A., Montoro P. (2009). Chemical analysis and quality control of *Ginkgo biloba* leaves, extracts, and phytopharmaceuticals. J. Chromatogr. A.

[2] Lin L.Z., Chen P., Ozcan M., Harnly J.M. (2008). Chromatographic profiles and identification of new phenolic components of *Ginkgo biloba* leaves and selected products. J. Agric. Food Chem..

[3] Vesna I., Mira P., Nada N., Mirjana R., Vesna V. (2008). The effect of *Ginkgo Biloba* (EGb 761) on epileptic activity in rabbits. Molecules.

[4] Hasler A., Sticher O., Meier B. (1990). High-performance liquid chromatographic determination of five widespread flavonoid aglycones. J. Chromatogr. A.

[5] Hasler A., Sticher O. (1992). Identification and determination of the flavonoids from *Ginkgo biloba* by high-performance liquid chromatography. J. Chromatogr. A.

[6] Van Beek T.A. (2002). Chemical analysis of *Ginkgo biloba* leaves and extracts. J. Chromatogr. A.

[7] Yao X., Shang E.X., Zhou G.S., Tang Y.P., Guo S., Su S.L., Jin C., Qian D.W., Qin Y., Duan J.A. (2012). Comparative characterization of total flavonol glycosides and terpene lactones at different ages, from different cultivation sources and genders of *Ginkgo biloba* leaves. Int. J. Mol. Sci..

[8] Wang C.G., Dai Y., Li D.L., Ma K.Y. (2010). *Ginkgo biloba* leaf extract action in scavenging free radicals and reducing mutagenicity and toxicity of cigarette smoke *in vivo*. J. Environ. Sci. Health A Tox. Hazard. Subst. Environ. Eng..

[9] Hyun S.K., Jung H.A., Chung H.Y., Choi J.S. (2006). *In vitro* peroxynitrite scavenging activity of 6-hydroxykynurenic acid and other flavonoids from *Gingko biloba* yellow leaves. Arch. Pharm. Res..

[10] Ou H.C., Lee W.J., Lee I.T., Chiu T.H., Tsai K.L., Lin C.Y., Sheu W.H. (2009). *Ginkgo biloba* extract attenuates oxLDL-induced oxidative functional damages in endothelial cells. J. Appl. Physiol..

[11] Trompezinski S., Bonneville M., Pernet I., Denis A., Schmitt D., Viac J. (2010). *Gingko biloba* extract reduces VEGF and CXCL-8/IL-8 levels in keratinocytes with cumulative effect with epigallocatechin-3-gallate. Arch. Dermatol. Res..

[12] Shi C., Zhao L., Zhu B., Li Q., Yew D.T., Yao Z., Xu J. (2009). Dosage effects of EGb761 on hydrogen peroxide-induced cell death in SH-SY5Y cells. Chem. Biol. Interact..

